# Low plasma concentrations of apolipoprotein M are associated with disease activity and endothelial dysfunction in systemic lupus erythematosus

**DOI:** 10.1186/s13075-019-1890-2

**Published:** 2019-05-02

**Authors:** Helena Tydén, Christian Lood, Andreas Jönsen, Birgitta Gullstrand, Robin Kahn, Petrus Linge, Sunil B. Kumaraswamy, Björn Dahlbäck, Anders A. Bengtsson

**Affiliations:** 1 Department of Rheumatology, Clinical Sciences, Lund University, SE-22185 Lund, Sweden; 2 Department of Pediatrics, Clinical Sciences Lund, Lund University, Lund, Sweden; 3 Wallenberg Centre for Molecular Medicine, Lund University, Lund, Sweden; 4 Department of Translational Medicine, Lund University, 214 28 Malmö, Sweden

**Keywords:** Systemic lupus erythematosus, Apolipoprotein M, Disease activity, Endothelial dysfunction

## Abstract

**Background:**

Apolipoprotein M (apoM) is a 25-kDa apolipoprotein present in 5% of high-density lipoprotein (HDL) particles. It is suggested to be anti-atherogenic and to play a key role in sustaining endothelial barrier integrity. SLE patients have increased cardiovascular disease risk, and we aimed to investigate if apoM levels reflect endothelial function in SLE. Since apoM plasma levels decrease during inflammatory conditions, our aim was also to determine the impact of SLE disease activity on apoM plasma levels.

**Methods:**

Plasma concentrations of apoM were measured by ELISA in two patient groups with systemic lupus erythematosus (SLE) and in 79 healthy control individuals. In patient group I (*n* = 84), evaluation time points were selected with the objective to include a wide range of clinical and laboratory variables reflecting disease activity which was measured as SLEDAI. In patient group II consisting of 140 consecutive patients, endothelial function was measured by a finger plethysmograph. A low Reactive Hyperemia Index (RHI) value indicates endothelial dysfunction.

**Results:**

SLE patients had decreased levels of apoM compared to healthy controls (*p* < 0.01), with apoM levels correlating inversely with SLEDAI (*r* = − 0.31, *p* < 0.01) as well as with levels of CRP (*r* = − 0.26, *p* = 0.02) and positively with levels of C3 (*r* = 0.29, *p* < 0.01). ApoM levels were particularly low in patients with active disease from the kidney and skin and in patients with leukopenia or positive anti-dsDNA antibody test (*p* < 0.05). ApoM levels correlated with RHI values in young SLE patients (*r* = 0.32, *p* = 0.01), consistent with the important role of apoM in regulating endothelial integrity.

**Conclusions:**

ApoM levels may be regulated by SLE-related inflammatory processes and could be a marker of disease activity and endothelial dysfunction, in particular in young SLE patients. Further studies are needed to investigate the predictive value of apoM in the development of a cardiovascular disease.

**Electronic supplementary material:**

The online version of this article (10.1186/s13075-019-1890-2) contains supplementary material, which is available to authorized users.

## Background

Systemic lupus erythematosus (SLE) is an inflammatory, autoimmune disorder with multi-organ involvement. Disease manifestations vary from mild to severe, and autoantibodies against nuclear antigens as well as autoreactive B and T lymphocytes play a prominent role in the pathogenesis. Endothelial dysfunction, which occurs early during atherosclerosis development, is able to predict subsequent cardiovascular disease (CVD) as well as premature atherosclerosis in SLE [1–4]. Endothelial function of the peripheral arteries can be assessed by several non-invasive methods, and flow-mediated dilatation (FMD) measurement of the brachial artery is considered the gold standard [5]. Another, easier way is to measure endothelial function in the finger arteries using a finger plethysmograph, Endopat [6]. In a previous study, a linear relationship between FMD and Endopat has been found [7]. Pathological values of brachial FMD and Endopat have been demonstrated to be independent predictors of cardiovascular disease [8]. The increased prevalence of CVD seen in SLE can to some extent be explained by atherosclerosis, but may also be due to other mechanisms [9–11]. Storage of lipids in the artery wall is considered a key event in the pathogenesis of atherosclerosis, and elevated plasma concentration of low-density lipoprotein (LDL) cholesterol is one of the main atherosclerosis risk factors. Further, the immune system and inflammatory processes play important roles in development of atherosclerosis [12, 13]. High-density lipoprotein (HDL) cholesterol consists of a variety of lipoproteins that exerts atheroprotective effects by different mechanisms [14]. Plasma HDL levels are measured in clinical routine in SLE patients and taken into account when scoring cardiovascular risk. It is also a tool when evaluating CVD risk in the general population, and lower levels indicate an elevated CVD risk [15]. The 25-kDa apolipoprotein M (apoM) is mainly associated with HDL and is present in about 5% of HDL particles [16]. ApoM is the physiological carrier of sphingosine-1-phosphate (S1P), a lipid mediator with vasculoprotective function that accounts for some of the anti-atherogenic properties of HDL [17]. In plasma, the major part of S1P is transported by the apoM-containing HDL particles [18]. ApoM delivers S1P to its receptors on endothelial cells where S1P-binding promotes a sealed endothelial barrier [18].

Systemic inflammation modulates protein and lipid composition of HDL, leading to changes in HDL function and impaired vasculoprotective effects [19, 20]. Furthermore, apoM levels decrease during the acute phase reaction [21]. We hypothesize that SLE-related inflammation may lead to lower plasma apoM levels that might contribute to endothelial dysfunction. The objectives of this study were to investigate if plasma concentrations of apoM were lower in active disease and if there was a difference in apoM levels during different kinds of organ system involvement. Additionally, we aimed to investigate endothelial function in SLE patients in relation to plasma levels of apoM.

## Methods

### Patients and healthy control subjects

The SLE patients in this study, all meeting four or more American College of Rheumatology (ACR) 1982 classification criteria for SLE [22], were taking part in a prospective control program at the Department of Rheumatology, Skåne University Hospital, Lund, Sweden. As part of this program, plasma samples were drawn at most visits and stored in a biobank. Two SLE patient groups and 79 healthy control individuals were investigated. Patient group I included patients with SLE, *n* = 84, and plasma sampled between 1985 and 2005 were retrieved from the biobank. These samples were used to compare plasma apoM levels with healthy individuals and to analyze apoM levels in relation to disease activity. In patient group I, one plasma sample from each SLE patient was selected. Time points when plasma samples were drawn from the patients were selected with the aim of including active disease and representing a wide range of manifestations and also including a number of patients with no disease activity. Clinical and demographic data were obtained retrospectively from medical records, and disease activity was assessed by SLEDAI-2 K [23]. Patient group II included consecutive patients with SLE (*n* = 140), participating in studies related to cardiovascular disease during 2011–2012 at the Department of Rheumatology, Skåne University Hospital, Lund, Sweden. At study inclusion of patients in group II, all participants were investigated by a rheumatologist and questionnaires regarding smoking and medication were completed. Medical history of the patients was obtained retrospectively from medical records, and disease activity was assessed using SLEDAI-2 K [23].

To determine organ damage due to SLE disease, the Systemic Lupus International Collaborating Clinics/American College of Rheumatology Damage Index (SLICC/ACR-DI) score of the SLE patients was registered [24]. Disease activity in both SLE groups was assessed using SLEDAI-2 K [23]. Overnight fasting blood samples were drawn according to standard procedures at Skåne University Hospital Lund for determination of plasma lipids and apoM. Complement and autoantibodies as well as erythrocyte sedimentation rate (ESR), CRP, and leukocyte count were measured by routine analyses at the Division of Clinical Immunology and Transfusion Medicine and Division of Clinical Chemistry at Skåne University Hospital, Lund, Sweden. The plasma samples from the SLE patients and controls were stored at − 80 °C.

Traditional CVD risk factors; age, gender, hypertension (systolic blood pressure equal or higher than 140 mmHg at the time point of blood sampling or treatment due to high blood pressure), and plasma LDL and HDL cholesterol levels were monitored. In patient group II, endothelial function was assessed with Endopat and this patient group was used to investigate plasma apoM levels in relation to endothelial function.

### ApoM ELISA

The apoM ELISA was performed as previously described [25]. In brief, 96-well costar plates (Corning, Inc. Lowell, MA, USA) were coated with a monoclonal apoM antibody (mAb 58) before blocking. The samples were diluted in detergent-containing buffer and incubated overnight. The bound apoM was detected using a biotinylated apoM antibody (mAb42) and developed using HRP-conjugated streptavidin and subsequent 1, 2-phenylenediamine dihydrochloride (Dako, Glostrup, Denmark). The 490 nm absorbance was measured and related to a plasma standard curve containing known amounts of apoM.

### Determination of endothelial function

Endothelial function was assessed using a finger plethysmograph (EndoPAT 2000, Itamar Medical, Caesarea, Israel), according to the manufacturer’s protocol as previously described [3, 6, 26]. The cut-off for reactive hyperemia index (RHI) was set to 1.67 according to the manufacturer’s instructions (Itamar) [27–29]. The technique is non-invasive and operator-independent. Probes with sensors were placed on the subjects’ index fingers, and pulse wave amplitude (PWA) changes in the index fingers were registered and analyzed by the computer software (Itamar). PWA in the right finger artery was registered at baseline, during 5 min of suprasystolic cuff occlusion and during reactive hyperemia. PWA from the left index finger, not undergoing cuff occlusion, was registered as a control. RHI was calculated with a computerized algorithm, where a low-value indicates impaired endothelial function.

### Statistical analyses

SPSS Statistics version 24 (IBM Corporation Armonk, NY, USA) was used for all statistical analyses. *T* test was performed when comparing groups. When comparing groups in small samples (*n* < 20) with skewed data, Mann**-**Whitney *U* test was used. Spearman’s rank correlation test and Pearson’s correlation test were used for correlation analyses. Statistical association between Endopat RHI value and apoM concentrations was calculated with linear regression analysis. To adjust for CVD risk factors and treatment, multiple regression analysis was used. In a group with skewed data, Spearman’s rank correlation test was used. A *p* value < 0.05 was considered statistically significant.

## Results

### Patients and controls

An overview of the demographics and plasma apoM levels of the SLE patients in patient groups I and II and the healthy controls is presented in Table 1. An overview of the ACR classification criteria and organ damage index in patient group I and II is presented in Table 2. Patients in group I had more organ damage than patients in group II with median SLICC-DI score 2 vs 1, respectively (Table 2). Patients in group I (*n* = 84) had higher median SLEDAI-2 K score as compared to patients in group II (*n* = 140), 6 vs 2, respectively. The distribution of SLEDAI-2 K scores in the two SLE groups is presented in Table 3. The treatments used in the two SLE groups at the time point of blood sampling are presented in Table 4.

**Table 1:** Demographics and plasma apoM levels in SLE patients and healthy control subjects

Groups	Patient group I *n* = 84	Patient group II *n* = 140	Healthy controls *n* = 79	*p* ^a^
Age, median (min-max), years	42 (16–85)	48,5 (20–81)	47 (18–81)	0.21
Gender, female (%)	88	86	85	0.54
Disease duration, median (min–max), years	7.5 (0–44)	11.5 (0–46)	–	–
apoM (μM), median (25, 75)	0.71 (0.52, 0.93)	0.81 (0.71, 1.02)	0.91 (0.80, 1.11)	< 0.01

*(25, 75)* 25th and 75th percentiles.- not applicable

^a^*P* value patient group I compared to healthy controls

Patient group I was used to compare plasma apoM levels with healthy controls

**Table 2:** ACR criteria and organ damage index in the two SLE patient groups

Groups	Patient group I	Patient group II
ACR criteria, median (min-max)	6 (4–10)	5 (4–10)
Malar rash (%)	67	52
Discoid rash (%)	37	20
Photosensitivity (%)	71	56
Oral ulcers (%)	27	26
Arthritis (%)	87	80
Serositis (%)	61	41
Renal disease (%)	42	34
Neurological disorder (%)	9	6
Hematological manifestations (%)	65	56
Leukopenia (%)	43	37
Lymphopenia (%)	33	26
Thrombocytopenia (%)	24	15
Immunology (%)	80	71
Anti-dsDNA antibodies (%)	74	61
Anti-Smith antibodies (%)	8	9
ANA (%)	99	98
SLICC-DI score^a^, median (min–max)	2 (0–9)	1 (0–8)

^a^Organ damage index

**Table 3:** Disease activity measured by SLEDAI-2 K score in the SLE patients at time-point of blood sampling

Groups	Patient group I *n* = 84	Patient group II *n* = 140
SLEDAI-2 K score, median (min–max)	6 (0–32)	2.0 (0–18)
Seizures (%)	1.2	0
Psychosis (%)	0	0
Organic brain syndrome (%)	0	0.7
Visual disturbance (%)	2.4	0
Cranial nerve disorder (%)	1.2	0
Lupus headache (%)	1.2	2.1
Cerebrovascular accident (CVA) (%)	3.6	0
Vasculitis (%)	2.4	0.7
Arthritis (%)	23.8	10.0
Myositis (%)	2.4	0
Kidney involvement (urinary cast, hematuria, proteinuria, or pyuria) (%)	25.0	10.7
Rash (%)	34.5	11.4
Oral or nasal ulcers (%)	2.4	2.1
Pleurisy (%)	4.8	0.7
Pericarditis (%)	4.8	0
Low complement (C3 or C4) (%)	44.0	25.0
Anti-dsDNA antibodies (%)	28.6	12.1
Fever (%)	10.7	0
Thrombocytopenia (%)	6.0	1.4
Leukopenia (%)	15.5	6.4
Alopecia (%)	9.5	2.1

In patient group I, plasma samples were drawn at time points of higher disease activity to investigate plasma apoM levels in relation to disease activity. In patient group II, plasma samples were drawn in consecutive patients to investigate apoM levels in relation to endothelial function. Individual items in SLEDAI-2 K score are shown

SLEDAI-2 K score was lower in patient group II

**Table 4:** Treatment in the SLE patients at time-point of blood sampling

Groups	Patient group I *n* = 84	Patient group II *n* = 140
Glukocorticoid dose ≤ 20 mg (*n*)	38	91
Glucocorticoid dose > 20 mg (*n*)	20	1
Antimalarial treatment (*n*)	43	100
Azathioprine (*n*)	18	31
Mycophenolatmofetil (*n*)	1	19
Intravenous immunoglobulin disorder (*n*)	3	2
Cyclophosphamide (*n*)	3	0
Cyclosporine (*n*)	5	2
Methotrexate (*n*)	0	13

### Plasma concentration of apoM is decreased in SLE and related to markers of inflammation and disease activity

**Fig. 1:**
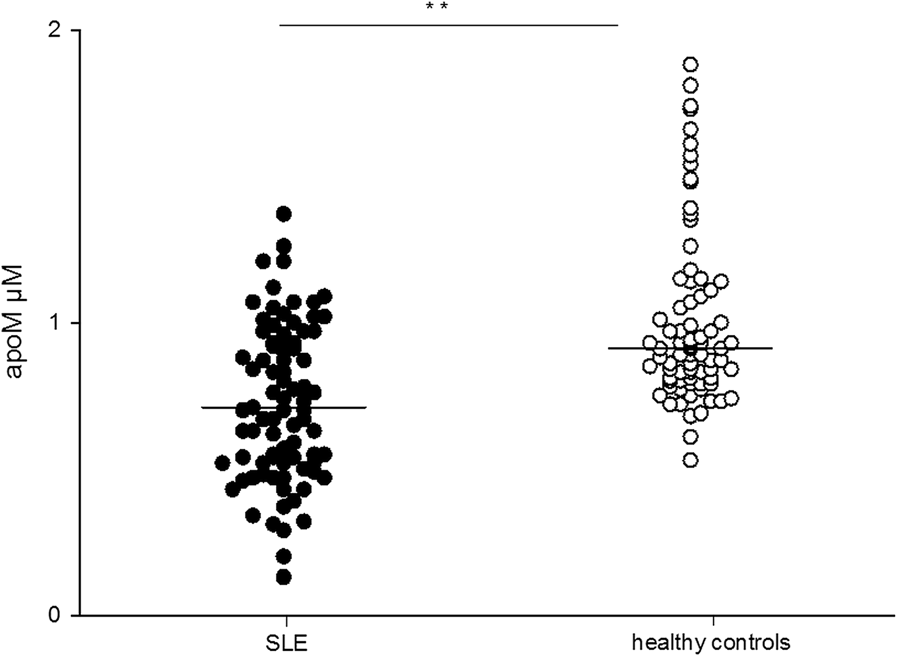
Comparison of plasma apoM levels in SLE patients and healthy control individuals. Decreased plasma apoM levels were seen in SLE patients compared to healthy control individuals

**Fig. 2:**
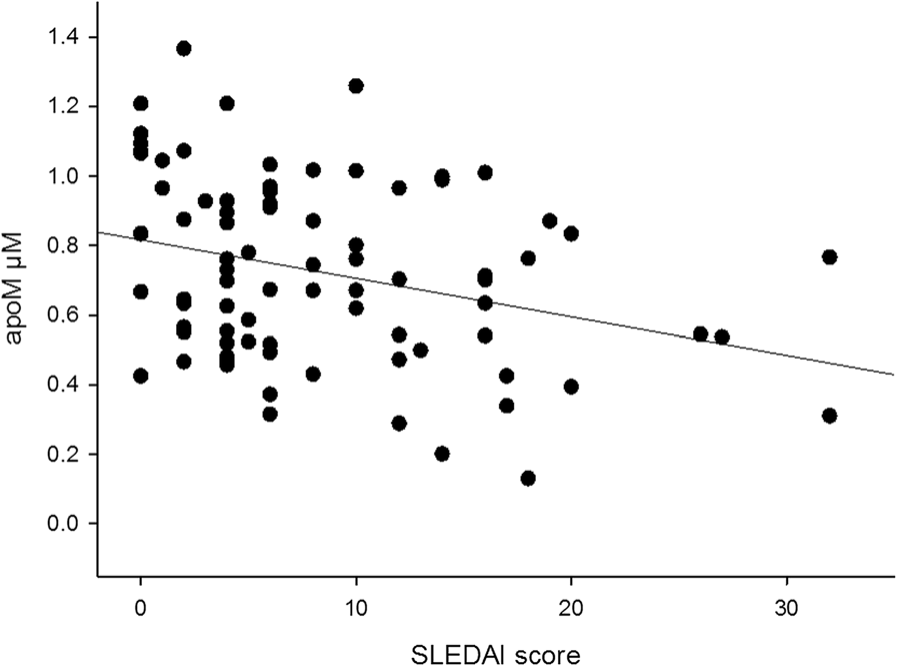
Inverse correlation between plasma apoM levels and SLEDAI score in SLE patients

Plasma apoM concentrations were significantly lower in SLE patients (patient group I, *n* = 84) compared to healthy individuals (*p* < 0.01, Fig. 1). ApoM levels correlated inversely to total SLEDAI-2 K score, (*r* = − 0.31, *p* < 0.01, Fig. 2) using Pearson’s correlation test. There was a negative correlation between apoM and CRP using Spearman’s correlation test (*r* = − 0.26, *p* = 0.02). This was not seen for ESR (Pearson’s correlation test). ApoM levels correlated with C3 serum levels (*r* = 0.29, *p* < 0.01, Additional file 1: Figure S1) (Pearson’s correlation test). No correlation was found between plasma apoM concentrations and C4, C1q, leukocyte, or platelet count.

### ApoM levels are associated with certain organ manifestations

**Fig. 3:**
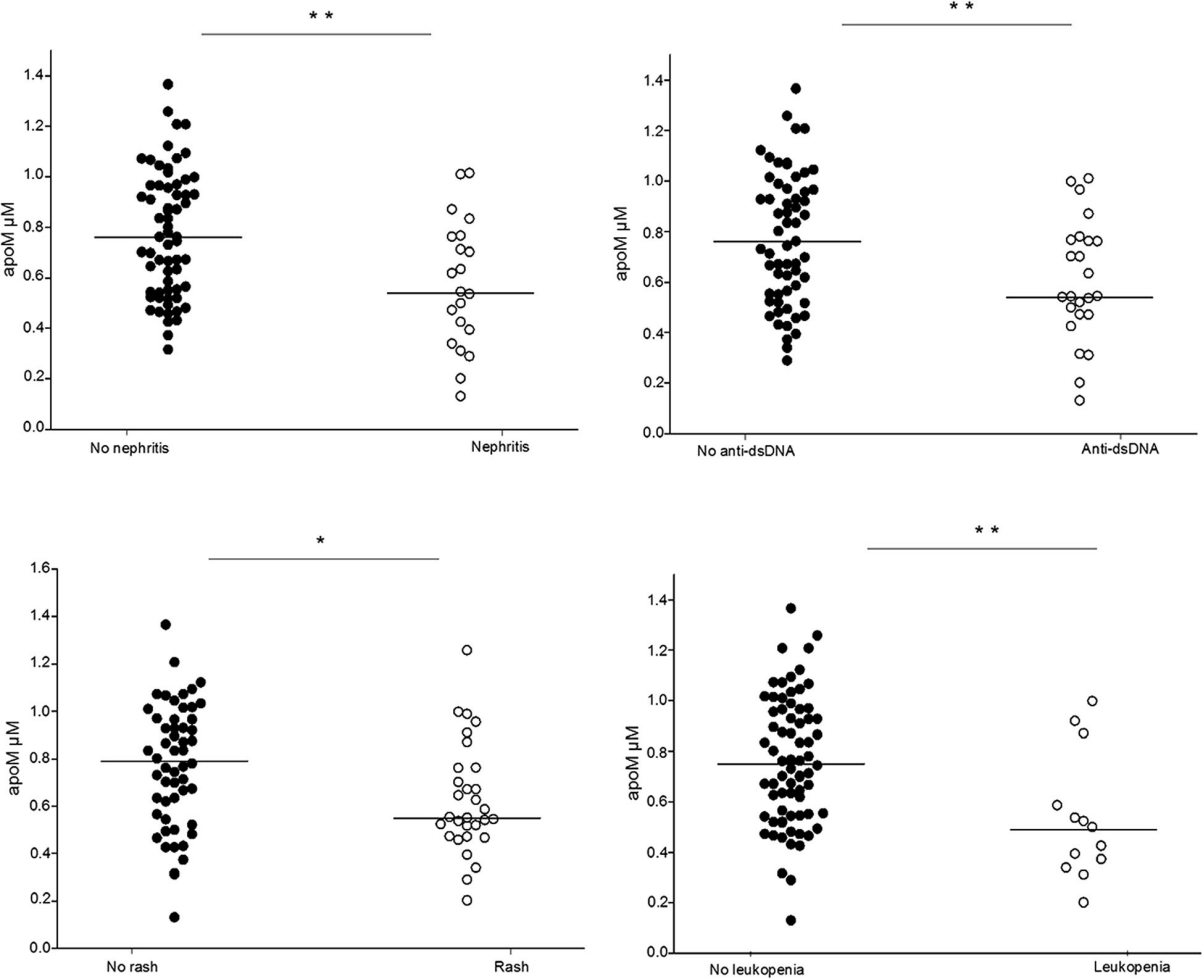
Comparison of plasma apoM levels in patients with and without the individual SLEDAI items, kidney involvement (urinary casts, proteinuria, hematuria, or pyuria), rash, and presence of anti-dsDNA antibodies and leukopenia. Decreased apoM levels were seen in SLE patients with disease activity in kidney and skin

Since several pathogenic pathways may be operative in various organ involvement during active disease, we investigated if apoM concentrations differed between organ system manifestations in SLE. In patient group I, apoM concentrations were significantly lower in patients with active renal disease (glomerulonephritis, e.g., urinary casts, proteinuria, hematuria, or pyuria) and skin involvement (rash), compared to patients without these organ manifestations (*p* < 0.01 and *p* = 0.01, respectively, Fig. 3). Furthermore, patients with the SLEDAI-2 K item leukopenia, or presence of anti-dsDNA antibodies at the time point of blood sampling, had decreased apoM levels compared to patients with normal leukocyte count, or absence of anti-dsDNA antibodies (*p* < 0.01, Fig. 3). Plasma concentrations of apoM in the different subgroups in patient group I are presented in Table 5.

**Table 5:** Plasma levels of apoM in SLE patients with disease activity in different organ systems

SLEDAI-2 K items^a^	apoM	n+	apoM	n−	*p*
Arthritis	0.82	20	0.70	64	0.37
Glomerulonephritis^b^	0.55	21	0.76	63	< 0.01
Rash	0.55	29	0.80	55	0.01
Low complement	0.67	37	0.75	46	0.06
Anti-dsDNA antibodies	0.54	24	0.78	60	< 0.01
Fever	0.59	9	0.73	75	0.33
Thrombocytopenia	0.92	5	0.70	79	0.73
Leukopenia	0.50	13	0.76	71	< 0.01
Alopecia	0.66	8	0.72	76	0.78

Median plasma concentrations (μM) of apoM in SLE patients in patients group I (*n* = 84) with (n+) and without (n−) different items in SLEDAI-2 K

^a^Only items with *n* ≥ 5 included

^b^Urinary casts, proteinuria, hematuria, or pyuria

Plasma apoM levels in patients with more than one organ manifestation at time point of blood sampling were analyzed. Patients with concurrent nephritis and rash (*n* = 8) had significantly lower apoM levels compared to patients with concurrent arthritis and rash (*n* = 7), *p* = 0.01. Values for patients with concurrent active arthritis and nephritis could not be calculated (*n* = 1). Median plasma apoM concentrations in these groups are demonstrated in Table 6.

**Table 6:** ApoM plasma levels in SLE patients with disease activity in more than one organ system

SLEDAI-2 K items	apoM	*n*
Rash + arthritis	0.87	7
Rash + nephritis	0.43	8
Nephritis + arthritis	0.77	1

Median plasma concentrations (μM) of apoM in SLE patients in patients group I (*n* = 84) with more than one item in SLEDAI-2 K. ApoM levels were significantly lower in patients with both skin rash and nephritis compared to patients with skin rash and arthritis, *p* = 0.01

### Plasma apoM levels are related to endothelial function

As apoM has endothelial barrier protective functions, we hypothesized that the lower apoM levels observed in SLE patients may account for an impaired endothelial function in those patients. To investigate this, patients within patient group II (*n* = 140) were examined cross-sectionally for endothelial function using Endopat.

**Fig. 4:**
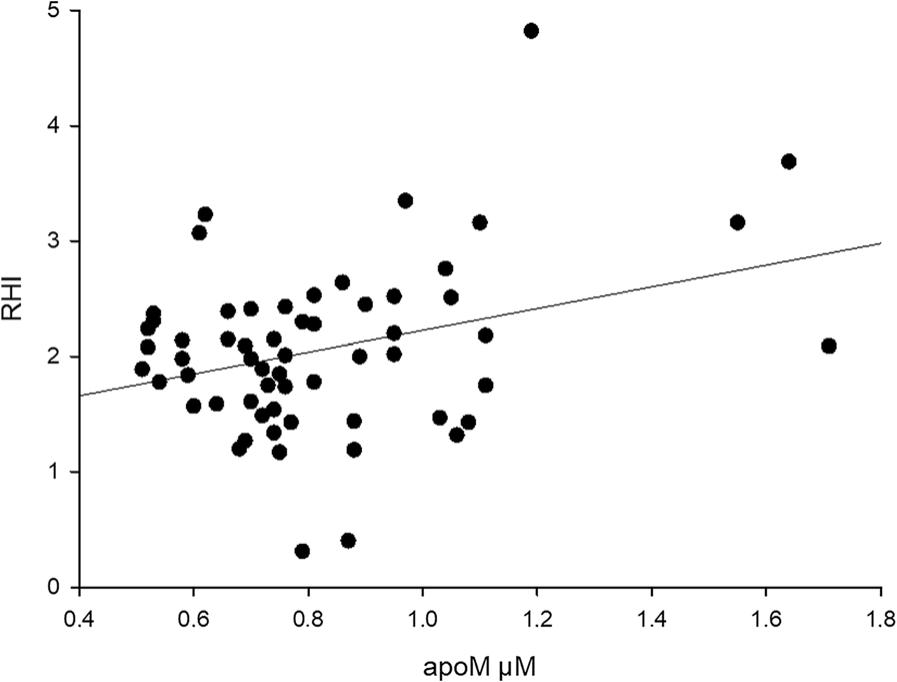
Correlation between endothelial function measured as reactive hyperemia index (RHI) and plasma apoM concentrations in SLE patients aged 20–45 years

Subgroup analyses of the younger patients (20–45 years of age) were performed since the most increased relative CVD risk is seen in this age group in SLE [9]. Using linear regression analysis, there was an association between RHI and apoM levels (*r* = 0.32, *p* = 0.01, beta = 0.94 95% CI 0.22–1.67) in SLE patients aged 20–45 years (*n* = 60). This association remained when adjusting for CVD risk factors (age, gender, hypertension, smoking, P-HDL) and treatment (blood lipid-lowering medication, e.g., statins, antimalarial drugs, e.g., hydroxychloroquine, and glucocorticoids) (*r* = 0.48, *p* < 0.01, beta = 1.26 95% CI 0.48–2.04, Fig. 4). The association also remained after adjusting for acute phase reaction (CRP) and SLE disease activity (SLEDAI scores) (*r* = 0.50, *p* < 0.01, beta = 1.33 95% CI 0.49–2.17, and *r* = 0.40, *p* < 0.01, beta = 1.04 95% CI 0.32–1.76, respectively, Fig. 4).

In patients 20–45 years of age, no association was seen between RHI and plasma concentrations of HDL or LDL (*r* = − 0.04, *p* = 0.76, beta = − 0.08, 95% CI − 0.58–0.43, and *r* = 0.25, *p* = 0.07, beta = 0.18, 95% CI − 0.02–0.38, respectively), suggesting that apoM is a superior marker of endothelial dysfunction in young SLE patients. When investigating SLE patients in group II, aged 46–81 years (*n* = 80), no association between apoM levels and RHI was seen (*r* = − 0.06, *p* = 0.59) using Spearman’s correlation test.

## Discussion

In this study, we demonstrate decreased plasma apoM concentrations in SLE patients with active disease, most pronounced in patients with renal and skin involvement, and in patients with presence of anti-dsDNA antibodies. Furthermore, in young SLE patients at marked risk of cardiovascular morbidity, apoM levels were associated with impaired endothelial function.

ApoM is the carrier of sphingosine-1-phosphat (S1P), an extracellular signaling molecule that regulates endothelial barrier function [30]. Since a role of apoM-S1P complex in prevention of endothelial dysfunction and atherosclerosis has been suggested [31, 32], we wanted to investigate if lower apoM levels in SLE could contribute to endothelial dysfunction. Therefore, we investigated endothelial function in SLE in relation to plasma apoM concentrations. We found that young SLE patients, 20–45 years of age, with lower levels of the vasculoprotective apoM had indeed lower RHI, indicating impaired endothelial function in this age group. The association remained also after adjusting for traditional CVD risk factors, plasma HDL and treatment with antimalarial drugs, glucocorticoids, and lipid-lowering medication. Thus, measuring apoM plasma levels, in particularly in young SLE patients, may add clinically important information about endothelial function and indicate which patients to monitor closer. A limitation of our study is the lack of mechanistic evidence for any causality of our finding that low apoM levels are associated with endothelial dysfunction in SLE specifically. However, several other mechanistic studies show favorable effects of apoM on the endothelium [18, 31].

The reason for low apoM levels in SLE has not been investigated previously. Dyslipidemia is well described in SLE, and elevated levels of triglycerides, very low-density lipoprotein cholesterol, and decreased levels of HDL are thought to contribute to the atherosclerotic development [33]. The changes in lipid composition observed in SLE patients may partly stem from SLE-related inflammation increasing secretory phospholipase [34] as well as reducing the overall activity of the HDL-bound antioxidant paraoxonase [35], thus affecting the lipid and protein content of HDL transforming it into a more pro-inflammatory lipoprotein, while losing its antioxidant effect [35, 36]. Further, apolipoprotein A-1, the main protein in HDL with capacity to reverse cholesterol transport, is replaced by serum amyloid A in inflammatory reactions including SLE, and may explain some of the impaired HDL functions [37]. Some SLE patients also develop autoantibodies towards apolipoprotein A-1, further decreasing its capacity to support efficient cholesterol efflux [37, 38].

Also, changes in apoM levels are thought to contribute to the changed HDL properties during inflammation [21]. Data from humans and mice demonstrate that apoM is a negative acute phase response protein with decreased mRNA and circulating protein levels during infection and inflammation. These changes in protein expression during inflammation is partly mediated by cytokines including tumor necrosis factor α and interleukin 1, cytokines that are of importance in SLE pathogenesis [39]. Given the decreased HDL levels and changed HDL structure seen in SLE as well as the lowered apoM levels described in inflammation, we hypothesize that apoM levels would be decreased in SLE and affected by disease activity. In the current study, we found a correlation between low apoM levels and markers of inflammation, CRP, and decreased C3, the last-mentioned being an indicator of SLE-related inflammation and disease activity. Our findings are in concordance with our main hypothesis that SLE-related inflammation might lower apoM levels. Lower apoM levels were most pronounced in patients with rash and glomerulonephritis, the latter connected to central pathogenetic pathways in SLE together with presence of anti-dsDNA antibodies where lower levels of apoM were also seen. In patients with more than one organ manifestation at time point of blood sampling, those with both skin and renal involvement had significantly lower apoM levels than patients with skin and arthritis, indicating that disease mechanisms that lead to arthritis in SLE may influence apoM levels to a lesser extent.

In a previous study, higher total cholesterol and triglyceride levels were found in patients with lupus nephritis as compared to patients with other types of chronic kidney disease with corresponding renal function. Further, lupus nephritis patients more often have abnormal LDL concentrations than patients with chronic kidney disease, suggesting an SLE-related cause of dyslipidemia, not entirely explained by the chronic renal disease [40]. In a recent study, enhanced atherosclerosis measured as presence of carotid plaques were found in SLE patients with a history of glomerulonephritis [41]. Altogether, the decreased apoM levels in patients with active lupus nephritis seen in our study fit well with the previous findings of dyslipidemia and atherosclerosis in SLE patients with renal involvement.

## Conclusion

The inflammatory processes in SLE may lower plasma apoM levels. The impaired endothelial function seen in younger SLE patients with lower apoM levels strengthens the hypothesis that apoM is important for endothelial health and might be considered a potential novel marker of endothelial function and health in young SLE patients.

## Additional file

Additional file 1: **Figure S1.** Correlation between plasma apoM levels and serum levels of C3 in SLE patients. (TIF 49 kb)

## Abbreviations

ACR: American College of Rheumatology
anti-dsDNA: Anti-double-stranded DNA
ApoM: Apolipoprotein M
CRP: C-reactive protein
CVD: Cardiovascular disease
ESR: Erythrocyte sedimentation rate
HDL: High-density lipoprotein
LDL: Low-density lipoprotein
RHI: Reactive hyperemia index
S1P: Sphingosine − 1-phosphate
SLE: Systemic lupus erythematosus
SLEDAI: SLE disease activity index
SLICC/ACR-DI: Systemic Lupus International Collaborating Clinics/ACR-Damage Index

## References

[1] Haider YS, Roberts WC (1981). Coronary arterial disease in systemic lupus erythematosus; quantification of degrees of narrowing in 22 necropsy patients (21 women) aged 16 to 37 years. Am J Med.

[2] Asanuma Y, Oeser A, Shintani AK, Turner E, Olsen N, Fazio S (2003). Premature coronary-artery atherosclerosis in systemic lupus erythematosus. N Engl J Med.

[3] Bonetti PO, Lerman LO, Lerman A (2003). Endothelial dysfunction: a marker of atherosclerotic risk. Arterioscler Thromb Vasc Biol.

[4] El-Magadmi M, Bodill H, Ahmad Y, Durrington PN, Mackness M, Walker M (2004). Systemic lupus erythematosus: an independent risk factor for endothelial dysfunction in women. Circulation.

[5] Anderson TJ (1999). Assessment and treatment of endothelial dysfunction in humans. J Am Coll Cardiol.

[6] Rubinshtein R, Kuvin JT, Soffler M, Lennon RJ, Lavi S, Nelson RE (2010). Assessment of endothelial function by non-invasive peripheral arterial tonometry predicts late cardiovascular adverse events. Eur Heart J.

[7] Kuvin JT, Patel AR, Sliney KA, Pandian NG, Sheffy J, Schnall RP (2003). Assessment of peripheral vascular endothelial function with finger arterial pulse wave amplitude. Am Heart J.

[8] Xu Y, Arora RC, Hiebert BM, Lerner B, Szwajcer A, McDonald K (2014). Non-invasive endothelial function testing and the risk of adverse outcomes: a systematic review and meta-analysis. Eur Heart J Cardiovasc Imaging.

[9] Manzi S, Meilahn EN, Rairie JE, Conte CG, Medsger TA, Jansen-McWilliams L (1997). Age-specific incidence rates of myocardial infarction and angina in women with systemic lupus erythematosus: comparison with the Framingham Study. Am J Epidemiol.

[10] Urowitz MB, Ibanez D, Gladman DD (2007). Atherosclerotic vascular events in a single large lupus cohort: prevalence and risk factors. J Rheumatol.

[11] Petri M, Perez-Gutthann S, Spence D, Hochberg MC (1992). Risk factors for coronary artery disease in patients with systemic lupus erythematosus. Am J Med.

[12] Ross R (1999). Atherosclerosis is an inflammatory disease. Am Heart J.

[13] Libby P, Ridker PM, Hansson GK (2009). Inflammation in atherosclerosis: from pathophysiology to practice. J Am Coll Cardiol.

[14] Nofer JR (2008). High-density lipoprotein, sphingosine 1-phosphate, and atherosclerosis. J Clin Lipidol.

[15] Emerging Risk Factors C, Di Angelantonio E, Sarwar N, Perry P, Kaptoge S, Ray KK (2009). Major lipids, apolipoproteins, and risk of vascular disease. JAMA.

[16] Xu N, Dahlback B (1999). A novel human apolipoprotein (apoM). J Biol Chem.

[17] Sattler K, Levkau B (2009). Sphingosine-1-phosphate as a mediator of high-density lipoprotein effects in cardiovascular protection. Cardiovasc Res.

[18] Christoffersen C, Obinata H, Kumaraswamy SB, Galvani S, Ahnstrom J, Sevvana M (2011). Endothelium-protective sphingosine-1-phosphate provided by HDL-associated apolipoprotein M. Proc Natl Acad Sci U S A.

[19] Yu BL, Wang SH, Peng DQ, Zhao SP (2010). HDL and immunomodulation: an emerging role of HDL against atherosclerosis. Immunol Cell Biol.

[20] Sattar N, Petrie JR, Jaap AJ (1998). The atherogenic lipoprotein phenotype and vascular endothelial dysfunction. Atherosclerosis.

[21] Feingold KR, Shigenaga JK, Chui LG, Moser A, Khovidhunkit W, Grunfeld C (2008). Infection and inflammation decrease apolipoprotein M expression. Atherosclerosis.

[22] Tan EM, Cohen AS, Fries JF, Masi AT, McShane DJ, Rothfield NF (1982). The 1982 revised criteria for the classification of systemic lupus erythematosus. Arthritis Rheum.

[23] Gladman DD, Ibanez D, Urowitz MB (2002). Systemic lupus erythematosus disease activity index 2000. J Rheumatol.

[24] Gladman D, Ginzler E, Goldsmith C, Fortin P, Liang M, Urowitz M (1996). The development and initial validation of the Systemic Lupus International Collaborating Clinics/American College of Rheumatology damage index for systemic lupus erythematosus. Arthritis Rheum.

[25] Axler O, Ahnstrom J, Dahlback B (2007). An ELISA for apolipoprotein M reveals a strong correlation to total cholesterol in human plasma. J Lipid Res.

[26] Goor DA, Sheffy J, Schnall RP, Arditti A, Caspi A, Bragdon EE (2004). Peripheral arterial tonometry: a diagnostic method for detection of myocardial ischemia induced during mental stress tests: a pilot study. Clin Cardiol.

[27] Bonetti PO, Pumper GM, Higano ST, Holmes DR, Kuvin JT, Lerman A (2004). Noninvasive identification of patients with early coronary atherosclerosis by assessment of digital reactive hyperemia. J Am Coll Cardiol.

[28] Peled N, Shitrit D, Fox BD, Shlomi D, Amital A, Bendayan D (2009). Peripheral arterial stiffness and endothelial dysfunction in idiopathic and scleroderma associated pulmonary arterial hypertension. J Rheumatol.

[29] Orabona R., Sciatti E., Vizzardi E., Bonadei I., Valcamonico A., Metra M., Frusca T. (2017). Endothelial dysfunction and vascular stiffness in women with previous pregnancy complicated by early or late pre-eclampsia. Ultrasound in Obstetrics & Gynecology.

[30] Blaho VA, Hla T (2014). An update on the biology of sphingosine 1-phosphate receptors. J Lipid Res.

[31] Ruiz M, Frej C, Holmer A, Guo LJ, Tran S, Dahlback B (2017). High-density lipoprotein-associated apolipoprotein M limits endothelial inflammation by delivering sphingosine-1-phosphate to the sphingosine-1-phosphate receptor 1. Arterioscler Thromb Vasc Biol.

[32] Christoffersen C, Jauhiainen M, Moser M, Porse B, Ehnholm C, Boesl M (2008). Effect of apolipoprotein M on high density lipoprotein metabolism and atherosclerosis in low density lipoprotein receptor knock-out mice. J Biol Chem.

[33] Borba EF, Bonfa E (1997). Dyslipoproteinemias in systemic lupus erythematosus: influence of disease, activity, and anticardiolipin antibodies. Lupus.

[34] de Beer FC, Connell PM, Yu J, de Beer MC, Webb NR, van der Westhuyzen DR (2000). HDL modification by secretory phospholipase A(2) promotes scavenger receptor class B type I interaction and accelerates HDL catabolism. J Lipid Res.

[35] Van Lenten BJ, Hama SY, de Beer FC, Stafforini DM, McIntyre TM, Prescott SM (1995). Anti-inflammatory HDL becomes pro-inflammatory during the acute phase response. Loss of protective effect of HDL against LDL oxidation in aortic wall cell cocultures. J Clin Invest.

[36] Khovidhunkit W, Kim MS, Memon RA, Shigenaga JK, Moser AH, Feingold KR (2004). Effects of infection and inflammation on lipid and lipoprotein metabolism: mechanisms and consequences to the host. J Lipid Res.

[37] Abe H, Tsuboi N, Suzuki S, Sakuraba H, Takanashi H, Tahara K (2001). Anti-apolipoprotein A-I autoantibody: characterization of monoclonal autoantibodies from patients with systemic lupus erythematosus. J Rheumatol.

[38] Delgado Alves J, Ames PR, Donohue S, Stanyer L, Nourooz-Zadeh J, Ravirajan C (2002). Antibodies to high-density lipoprotein and beta2-glycoprotein I are inversely correlated with paraoxonase activity in systemic lupus erythematosus and primary antiphospholipid syndrome. Arthritis Rheum.

[39] Baumann H, Prowse KR, Marinkovic S, Won KA, Jahreis GP (1989). Stimulation of hepatic acute phase response by cytokines and glucocorticoids. Ann N Y Acad Sci.

[40] Chong YB, Yap DY, Tang CS, Chan TM (2011). Dyslipidaemia in patients with lupus nephritis. Nephrology (Carlton).

[41] Gustafsson JT, Herlitz Lindberg M, Gunnarsson I, Pettersson S, Elvin K, Ohrvik J (2017). Excess atherosclerosis in systemic lupus erythematosus,-A matter of renal involvement: case control study of 281 SLE patients and 281 individually matched population controls. PLoS One.

