# Supplementary figures and images for: Low plasma concentrations of apolipoprotein M are associated with disease activity and endothelial dysfunction in systemic lupus erythematosus
Source: Arthritis Res Ther. 2019 May 2;21:110. doi: 10.1186/s13075-019-1890-2 (PMC6498515; doi:10.1186/s13075-019-1890-2)

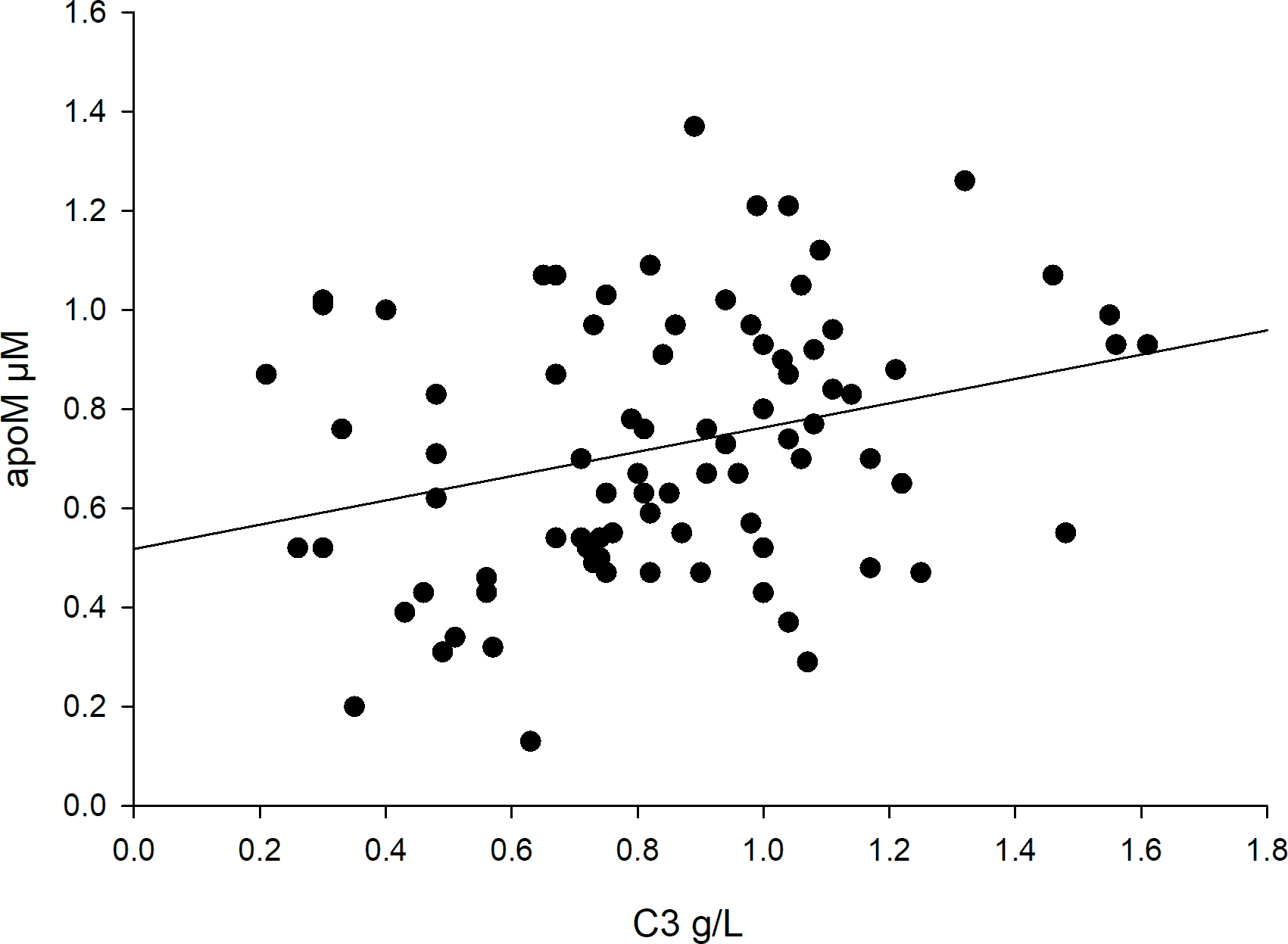

Supplement: Supplementary file 1 — Figure S1. Correlation between plasma apoM levels and serum levels of C3 in SLE patients. (TIF 49 kb) [file 13075_2019_1890_MOESM1_ESM.tif]
